# Wound-Induced Systemic Responses and Their Coordination by Electrical Signals

**DOI:** 10.3389/fpls.2022.880680

**Published:** 2022-05-18

**Authors:** Kyounghee Lee, Pil Joon Seo

**Affiliations:** ^1^Department of Chemistry, Seoul National University, Seoul, South Korea; ^2^Research Institute of Basic Sciences, Seoul National University, Seoul, South Korea; ^3^Plant Genomics and Breeding Institute, Seoul National University, Seoul, South Korea

**Keywords:** electrical signal, ion channel, ion pump, jasmonic acid, membrane potential, systemic signaling, wounding

## Abstract

Wounding not only induces the expression of damage-responsive genes, but also initiates physiological changes, such as tissue repair, vascular reconnection, and *de novo* organogenesis in locally damaged tissues. Wound-induced signals also propagate from the site of wounding to distal organs to elicit a systemic response. Electrical signaling, which is the most conserved type of systemic signaling in eukaryotes, is triggered by wound-induced membrane potential changes. Changes in membrane potential spread toward systemic tissues in synergy with chemical and hydraulic signals. Here, we review current knowledge on wound-induced local and systemic responses in plants. We focus particularly on how wound-activated plasma membrane-localized ion channels and pumps propagate systemic information about wounding to induce downstream molecular responses in distal tissues. Finally, we propose future studies that could lead to a better understanding of plant electrical signals and their role in physiological responses to wounding.

## Introduction

Plants are sessile organisms that are constantly exposed to a variety of external stimuli. Wounding is one external stimulus that induces physical damages threating plant survival and also makes them more susceptible to pathogen invasion (Savatin et al., 2014). Wounding is caused by insect feeding, pathogen infection, animal grazing, wind, and heavy rain (Reymond et al., 2000; Sozen et al., 2020). To survive, plants have evolved sophisticated defense mechanisms, such as the activation of defense gene expression, tissue repair, and *de novo* tissue regeneration in regions of the tissue where wounding occurs (Reymond et al., 2000; Liu et al., 2014; Savatin et al., 2014; Matsuoka et al., 2021). Signals in response to wounding propagate from the site of wound perception to distal tissues. These signals typically travel long distances, and this process is referred to as a systemic response (Huber and Bauerle, 2016). Wounding triggers distal systemic responses in plants, such as accumulation of defensive proteinase inhibitors (PIs) and jasmonates (JAs) in systemic tissues (Koo et al., 2009; Kiep et al., 2015; Gilroy et al., 2016; Fichman and Mittler, 2020).

Long-distance signals transduce environmental information throughout the whole plant body, and three major types of long-distance signals have been proposed: (i) chemical; (ii) hydraulic; and (iii) electrical signals (Huber and Bauerle, 2016; Canales et al., 2017). In particular, plant electrical signaling is the most conserved long-distance signaling system, which travels to plant distal tissues through vascular tissues to systemically induce responses in a whole plant (Hedrich et al., 2016). It is capable of transmitting signals more quickly over long distances, compared with hormone signals (Choi et al., 2017; Moe-Lange et al., 2021). Electrical signals in plants can be divided into several sub-types: local electrical potential (LEP), action potential (AP), systemic potential (SP), and slow wave potential (SWP; also known as variation potential; Szechynska-Hebda et al., 2017; Mudrilov et al., 2021). LEP is a sub-threshold response induced by change in environmental conditions, such as humidity light and temperatures. While LEP is only locally generated and is not transferred to other parts of a plant, AP, SP, and SWP can transmit from the stimulated site to other parts of the plant. AP can be defined as a rapid and transient changes in cell membrane potential (depolarization and subsequent repolarization) in response to non-damaging stimuli. AP induces an all-or-nothing response, which occurs only above a threshold of stimuli. In contrast, SWP defined as a transient membrane depolarization with an irregular shape and a longer and delayed repolarization phase is a hydraulically propagating electrical signal exclusively found in plant cells. SWP is usually induced by severe tissue damage. SP is a most recently proposed type that has variable intensities according to stimuli. SP displays a transient hyperpolarization that is usually induced by herbivore feeding or adding cations to a damaged tissues (Zimmermann et al., 2009, 2016; Szechynska-Hebda et al., 2017). To date, SWP has been extensively investigated in plants. SWP is characterized by a decrease in magnitude as it spreads away from the stimulated site, and magnitude and shape of SWP vary with the intensity of the environmental stimulus (Huber and Bauerle, 2016). Notably, wound-induced changes in membrane potential possibly generate a unique profile of electrical signals (Mousavi et al., 2014; Farmer et al., 2020), which is possibly shaped by wound-activated ion channels and pumps (Gadsby, 2009; Kumari et al., 2019; Farmer et al., 2020).

Here, we summarize current knowledge about local and systemic wound responses in plants. We also discuss how wound-activated ion channels and pumps generate and propagate electrical signals to induce downstream wound responses. Finally, we propose future studies that could help to identify the novel roles of electrical signaling upon wounding.

## Responses to Wounding

To mitigate the plant damages from wounding, plants not only trigger tissue healing and regeneration, but also activate multiple defense responses (Leon et al., 2001; Cheong et al., 2002). Signals that are generated by wounding are transmitted through long distances. The activation of defense genes in tissues located away from the site of wounding is a well-known systemic response (Huber and Bauerle, 2016). In this section, we summarize the cellular and molecular processes underlying local and systemic responses to wounding.

### Local Responses to Wounding

**Figure 1:**
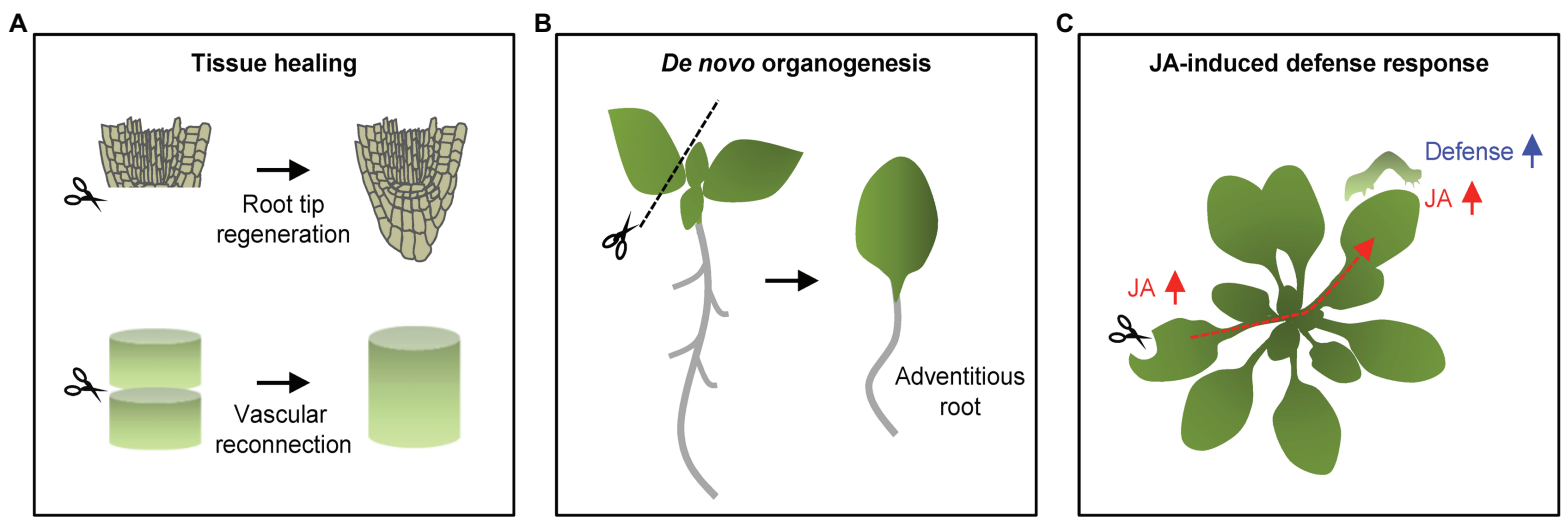
Wound-induced local and systemic responses in plants. **(A)** Wound healing and tissue regeneration. Upon wounding, plants stimulate wound healing and tissue regeneration. Excision of the root apical meristem and vascular tissues leads to the tissue restoration in local damaged regions. **(B)** Wound-induced adventitious root formation. Wounding occasionally results in cell fate changes as shown by the adventitious root formation from detached leaf explants. **(C)** Wound-induced defense responses. Wounding also increases defense capacity in both local and systemic tissues. Jasmonic acid (JA) is a pivotal phytohormone stimulating plant defense responses not only in damaged local tissues, but also distal plant organs. Long-distance signals propagate through vasculatures in order to confer systemic defense resistance in a whole plant.

The healing of injured tissue is a primary local response to wounding (Hoermayer and Friml, 2019; Marhava et al., 2019; Matsuoka et al., 2021). Upon wounding, plants rely on targeted cell division and expansion to restore their damaged tissues. For example, elimination of specific root cells by laser ablation promotes periclinal cell division of cells adjacent to the inner side of the injury site through the reactivation of stem cell transcriptional programs, ensuring the correct replacement of injured cells (Marhava et al., 2019). Moreover, excision of the root apical meristem triggers root tip regeneration *via* embryonic developmental process as well as hormone redistributions (Efroni et al., 2016). The root tip regeneration in *Arabidopsis* is known to rely on genetic program consisting of *PLETHORA 2* (*PLT2*) and *ETHYLENE RESPONSE FACTOR 115* (*ERF115;* Durgaprasad et al., 2019; Canher et al., 2020). The tissue repair is also pervasive in other tissues. Damage to vascular tissues leads to the formation of cambial cells from differentiated cells, which then re-differentiate into xylem and phloem vessels in an auxin-dependent manner (Sussex et al., 1972; Mazur et al., 2016; Matsuoka et al., 2021; Figure 1).

Wounding triggers neighboring cells to re-enter the cell cycle, frequently inducing callus from differentiated somatic cells (Ikeuchi et al., 2017; Iwase et al., 2021). The AP2/ERF transcription factor gene, *WOUND-INDUCED DEDIFFERENTIATION1* (*WIND1*), and its close homologs *WIND*2, *WIND3*, and *WIND4* are rapidly induced by wounding, and they promote callus formation by activating cytokinin responses (Iwase et al., 2011). Callus has the capacity for cell fate transition, which is essential for regenerating new organs (Iwase et al., 2021). In parallel, wounding frequently activates *de novo* organogenesis, sometimes independently of callus formation. For instance, cutting leaf explants triggers *de novo* root organogenesis (Liu et al., 2014). Up-regulation of JA biosynthesis and signaling subsequently stimulates auxin biosynthesis that precedes cell fate transition into root founder cell (Zhang et al., 2019; Figure 1).

Consistent with the fact that JA is a major wound-responsive phytohormone (Koo et al., 2009), JA-induced defense responses are mainly activated in local damaged tissues. JA biosynthesis is rapidly initiated within 30 s by 13-lipoxygenases (13-LOXs) in local damaged leaves, which catalyze the incorporation of an oxygen molecule to chloroplastic tri-unsaturated fatty acids (Glauser et al., 2009; Ahmad et al., 2016; Li et al., 2016; Figure 1). Following wound-induced increases in JA levels, the F-box protein, CORONATINE INSENSITIVE 1 (COI1), physically interacts with JASMONATE-ZIM DOMAIN (JAZ) proteins, which are repressors of JA signaling (Gfeller et al., 2010). JA-induced COI1–JAZ interaction triggers JAZ degradation through the ubiquitin-dependent pathway, de-repressing MYC2 transcription factor activity (Guo et al., 2018). MYC2 activates defense genes and therefore increases the capacity of plants to defend themselves against wounding as well as herbivory (Chauvin et al., 2013; Major et al., 2017).

### Systemic Responses to Wounding

Upon injury, wound-responsive genes are also induced in undamaged systemic tissues to protect plants against potential future damages. Accumulation of JA is essential to elicit defensive traits in systemic tissues. JA accumulation in systemic tissues significantly increases 120 s after wounding (Glauser et al., 2009). Among the four 13-LOXs, LOX6 contributes the most to JA biosynthesis in *Arabidopsis* systemic leaves (Chauvin et al., 2013). JA-inducible PIs protect plant systemic tissues from herbivores by reducing the activity of digestive enzymes in insect guts (Howe, 2004). In tomato, expression of *PI* genes is induced in systemic tissues within 2 h after wounding, and their induction is impaired in JA signaling mutants (Li et al., 2002). Application of methyl JA to one leaf induces *PI* gene expression in both local and systemic leaves even in the absence of wounding (Farmer and Ryan, 1990; Devoto and Turner, 2003).

Extracellular signals such as cell wall-derived oligogalacturonides (OGs) and peptides have been characterized as typical systemic signals of wounding, which are known as Damage-Associated Molecular Patterns (DAMPs). For instance, systemin, a 18-amino acid peptide, was identified in tomato after wounding or insect attack as a cleavage product released into the apoplast from the 200-amino acid precursor prosystemin protein (Sun et al., 2011). Systemin triggers systemic responses by acting upstream of JA (Sun et al., 2011). Ectopic expression of prosystemin triggers the constitutive expression of *PI* genes in the absence of wounding, and the increased *PI* levels are reduced in JA-deficient mutants (Sun et al., 2011). In addition to *PIs*, expression of *PATHOGENESIS-RELATED* (*PR*) genes, encoding PR-1, PR-10, chitinase, thaumatin-like protein, and plant defensin, is induced in systemic tissues to enhance plant survival and resistance against further herbivore attack (Kang et al., 2021).

## Long-Distance Signals in Response to Wounding

As noted, wound-induced defense responses propagate from injured local tissues to systemic tissues through long-distance signals. Long-distance signals are divided into three types: chemical, hydraulic, and electrical (Huber and Bauerle, 2016; Moe-Lange et al., 2021). Chemical signaling usually employs reactive oxygen species (ROS) and calcium ion (Ca^2+^; Moe-Lange et al., 2021). Wounding rapidly triggers the production of ROS in local and systemic tissues that is dependent on the NADPH oxidase, RESPIRATORY BURST OXIDASE HOMOLOG D (RBOHD; Miller et al., 2009; Prasad et al., 2019). Transgenic plants expressing luciferase driven by a ROS-responsive *ZAT12* gene promoter show that ROS signaling propagates into systemic tissues at a rate of ~8 cm/min, and this rate is substantially delayed in p*ZAT12::Luc/rbohD* plants (Miller et al., 2009). An increase in cytosolic Ca^2+^ is another hallmark of long-distance signaling (Shao et al., 2020). Wounding elevates cytosolic Ca^2+^ within seconds at the injured site, which generates a Ca^2+^ wave that travels into systemic tissues through vasculature tissues at rates of ~6 cm/min (Tian et al., 2020). The Glu-activated GLUTAMATE RECEPTOR-LIKE (GLR) ion channel family expressed in phloem and xylem is responsible for the increases in intracellular Ca^2+^ concentration in local and systemic tissues (Gilroy et al., 2016; Toyota et al., 2018). Notably, accumulation of Ca^2+^ is required for systemic JA induction, and consistently, blocking Ca^2+^ entry inhibits JA-induced defense responses (Tian et al., 2020). Moreover, hydraulic signaling is a class of long-distance signaling that is closely associated with initial Ca^2+^ entry into cells (Gilroy et al., 2016). Wounding causes rapid axial changes of hydrostatic pressure in the xylem and then, converts it to slower, radially dispersed changes of pressure associated with activation of a clade 3 GLR-dependent signaling pathway that prepares distal leaves for imminent attack (Farmer et al., 2014).

Electrical signaling is another crucial type of long-distance signaling generated by a rapid collapse of membrane potential in response to wounding (Farmer et al., 2020). Among several sub-types of electrical signals, SWP is particularly triggered by local wounding and transmitted to undamaged tissues at a speed of ~7 cm/min through vascular connections in plants (Mousavi et al., 2013; Szechynska-Hebda et al., 2017; Nguyen et al., 2018; Kurenda et al., 2019). Although waves of SWP have variable dynamics, it is generally characterized by a rapid (< 2 s) and massive (>50 mV) depolarization phase, and a slow (> 5 min) repolarization phase after wounding in *Arabidopsis* (Nguyen et al., 2018; Kumari et al., 2019). Notably, SWP has a diverse range of intensities, propagation speed and amplitude depending on the severity of the injury and distance from the injured site (Huber and Bauerle, 2016). The variable SWP dynamics are likely decoded into appropriate defense responses in plants.

Although the long distance signals are closely interconnected (Farmer et al., 2020), SWPs act as key early responses that initiate wound-induced systemic responses, which precede increases in cytosolic Ca^2+^ accumulation and JA biosynthesis in distal tissues (Vega-Munoz et al., 2020; Moe-Lange et al., 2021). Several plasma membrane-localized ion channels and pumps are characterized as core regulators in generating and propagating SWPs in response to wounding (Mousavi et al., 2013; Kumari et al., 2019; Moe-Lange et al., 2021).

### Glutamate Receptor-Like Channels

**Figure 2:**
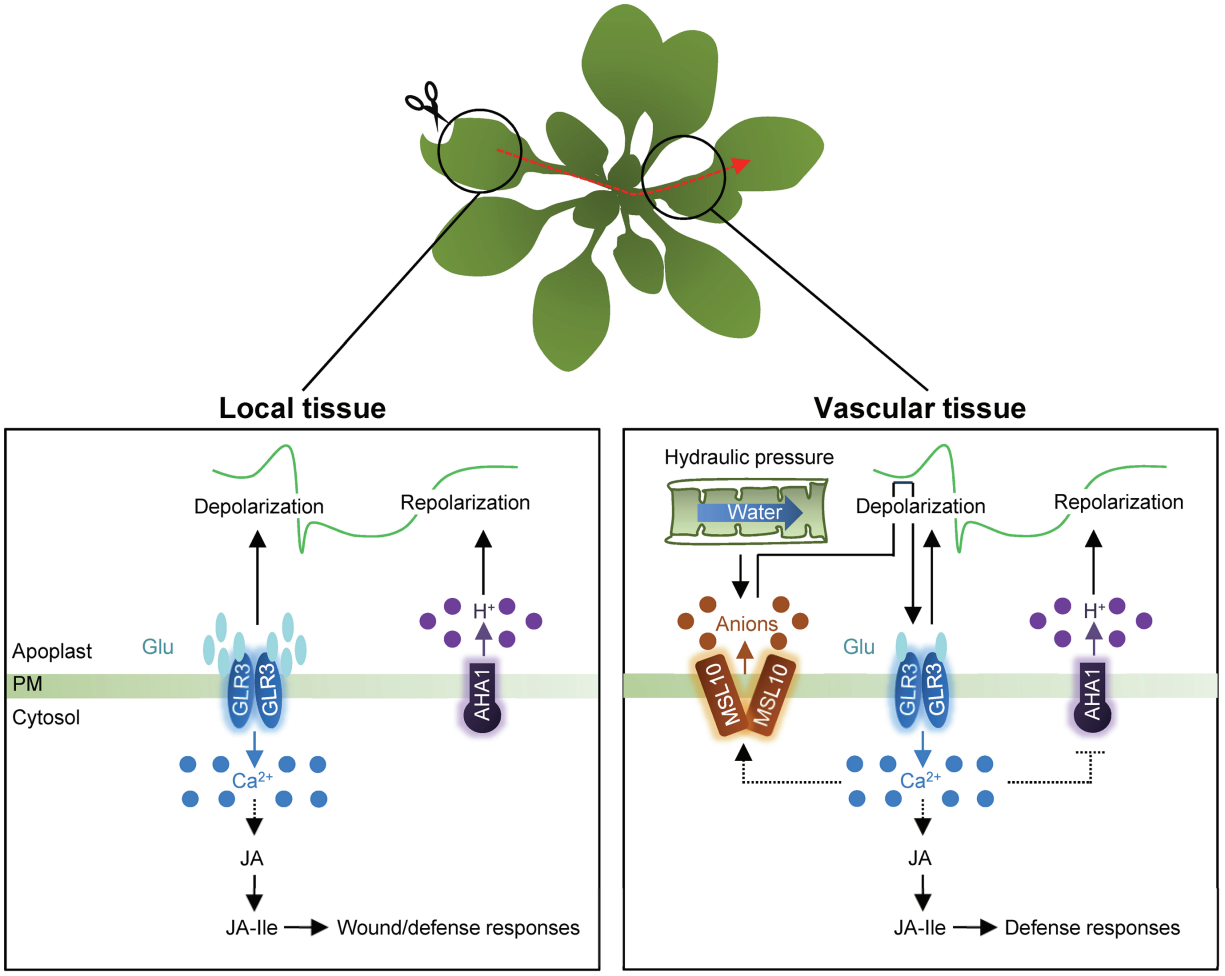
Schematic representation of electrical signaling in local and systemic tissues. In local tissue, wounding triggers an increase in apoplastic Glu concentration and then, induces GLR-dependent Ca^2+^ influx as well as depolarization of membrane potential. The GLR proteins likely act as tetramers. Following depolarization, H^+^-ATPase AHA1 is then required for membrane repolarization. The electrical signals systemically propagate through vasculature tissues in a connection with Ca^2+^ wave. Changes in turgor pressure affect membrane tension and activate the heptameric anion channel, MSL10, causing membrane depolarization through anion efflux. The membrane depolarization fully activates GLRs in response to wounding. GLR-dependent Ca^2+^ influx potentially causes activation of MSL10, simultaneously with the deactivation of AHA1. Synergistic propagation of Ca^2+^ waves and SWPs promotes JA biosynthesis to induce defense responses in systemic tissues. PM, plasma membrane.

The *GLR* genes encode putative glutamate receptors and Ca^2+^ permeable channels (Nguyen et al., 2018). The *Arabidopsis* genome contains 20 *GLR* genes that are divided into three clades (Ni et al., 2016). Among others, Clade 3 GLR members have key roles in the generation and propagation of SWP (Mousavi et al., 2013; Vega-Munoz et al., 2020). Upon wounding, the concentration of apoplastic Glu increases in local damaged tissues. The Glu excretion subsequently activates GLRs to induce Ca^2+^ influx, which is potentially linked to membrane depolarization (Mousavi et al., 2013; Toyota et al., 2018). In support, mutating clade 3 *GLR* genes, such as *GLR3.1*, *GLR3.2*, *GLR3.3*, or *GLR3.6*, reduces Ca^2+^ influx and also the duration of SWPs in local wounded leaves (Mousavi et al., 2013). Propagation of SWP is particularly dependent on two GLR members, GLR3.3 and GLR3.6, which are expressed in the phloem and xylem contact cells, respectively, as SWP propagation in distal leaves is abolished in *glr3.3 glr3.6* double mutants (Mousavi et al., 2013; Salvador-Recatala, 2016; Toyota et al., 2018; Li et al., 2021; Moe-Lange et al., 2021). Similarly, OsGLR3.4, which is a clade 3 GLR member in rice, also functions in wound-induced SWP propagation (Yu et al., 2022). Induction of SWPs in systemic leaves by mechanical stress in roots is significantly reduced in *osglr3.4* mutants, indicating the functional conservation of GLRs across diverse plant species (Yu et al., 2022). Given that the GLR ion channels stimulate an increase in intracellular Ca^2+^ concentration that propagates to distant organs, the intimate connection between electrical and Ca^2+^ signals synergistically leads to efficient activation of systemic responses (Figure 2).

Notably, different GLR members further fine-tune distribution of systemic electrical signals. GLR3.5 prevents the propagation of wound-induced electrical potentials to distal non-neighbor leaves. The *glr3.5* mutant propagates systemic electrical signal in non-neighbor leaves where wound-induced electrical signal is not transmitted in wild type (Salvador-Recatala, 2016). Collectively, electrical signaling is propagated throughout the plant, and distribution of the systemic information is elaborately regulated by GLR proteins.

### Phosphorylation (P)-Type Proton ATPase 1

P-type ATPases facilitate movement of protons, ions, and molecules across membranes, and determine membrane potential in animal kingdom (Kumari et al., 2019). Notably, plasma membrane-localized H^+^-ATPases within the P-type superfamily act as ion pumps and are involved in SWP propagation in plants (Falhof et al., 2016), and treatment with fusicoccin (FC), which activates plasma membrane H^+^ -ATPases, attenuates SWP duration (Kumari et al., 2019).

The *Arabidopsis* genome contains 11 members encoding H^+^-ATPases (AHA; Robertson et al., 2004; Haruta et al., 2010; Lin et al., 2021). AHA1 primarily functions in regulating SWPs in response to wounding (Kumari et al., 2019; Shao et al., 2020). In contrast to GLRs, AHA1 negatively controls SWP duration (Kumari et al., 2019; Figure 2). *aha1* mutants extend the duration of the repolarization phase of SWPs in both local and systemic tissues, while the *AHA1* gain-of-function mutant, *ost2-2D*, reduces SWP repolarization duration in systemic leaves (Kumari et al., 2019). Consistent with changes in the duration of SWPs, prolonged Ca^2+^ transients and JA biosynthesis increase in systemic tissues of *aha1* mutants (Kumari et al., 2019).

It should be noted that, GLRs and AHA1 may function in common pathways for SWP generation, as revealed by double mutant studies (Kumari et al., 2019). The duration of the repolarization phase of *aha1* mutants was lower than that of wild type in both local and systemic tissues when crossed with *glr3.3* mutants (Kumari et al., 2019). In addition, co-treatment with Glu and the H^+^-ATPase activating compound FC abolishes the amplitude and duration of SWP depolarization, demonstrating that constitutive activation of H^+^-ATPase blocks Glu-induced depolarization (Shao et al., 2020).

### Mechanosensitive Ion Channel-Like 10 (MSL10)

Mechanosensitive ion channels (MscS) respond to physical forces or plasma membrane deformation and generate mechanosensitive ion flux (Basu and Haswell, 2017; Canales Coutino and Mayor, 2021). MscS-Like (MSL) channels are the best-characterized MscS in diverse organisms (Basu and Haswell, 2017). Among 10 MSL proteins in *Arabidopsis* (Haswell et al., 2008), MSL10 is known to be directly gated by lateral membrane tension (Basu and Haswell, 2020) and has preference for anions with the conductance of about 100 pS (Basu and Haswell, 2020). Notably, the vasculature-expressed MSL10 protein functions in systemic SWP propagation upon wounding in *Arabidopsis* (Moe-Lange et al., 2021). The mutation of *MSL10* significantly reduces the duration of SWP in systemic leaves, which also leads to reduced Ca^2+^ waves and *JASMONATE-ZIM-DOMAIN PROTEIN 10* expression in distal tissues of *msl10* mutants (Moe-Lange et al., 2021), indicating that the mechanosensitive ion channel MSL10 is required for wound-elicited electrical signals and systemic Ca^2+^ wave in distal tissues (Figure 2).

The *msl10* mutants display ~fourfold shortened systemic SWPs and reduced Ca^2+^ wave in distal tissues, similar to the effects observed in the *glr3.1*, *glr3.3*, and *glr3.6* single mutants (Moe-Lange et al., 2021). Considering that not only the spatial expression of *GLR3.1*, *GLR3.3*, and *GLR3.6* overlaps with that of *MSL10* in vasculature tissues, but also reduced SWP magnitudes of *msl10;glr3.3* and *msl10;glr3.6* double mutants are indistinguishable from those of their single mutants, MSL10 and GLRs function in overlapping genetic pathways (Moe-Lange et al., 2021). Taken together, wounding triggers the release of glutamate into the apoplasm, in turn activating Ca^2+^-permeable GLR3s that also influence electrical propagation. In parallel, changes in turgor pressure affect membrane tension and activate MSL10, causing anion efflux and depolarization of membrane potential. Membrane depolarization promotes full activity of the GLRs in distal tissues in response to wounding. Synergistic propagation of Ca^2+^ waves and SWPs efficiently induces systemic wound responses for plant survival (Fromm and Lautner, 2007; Feng et al., 2021).

## Conclusion and Prospects

Plants have evolved long-distance communication system that systemically coordinates plant growth and development, especially enabling them to cope with adverse environments. Electrochemical signals are likely to mediate intercellular and intracellular communication in association with environmental changes. In this context, this study focused particularly on wound-responsive electrical signaling. Despite advances in understanding on the role of electrical signaling in systemic wound responses, several key questions still remain to be resolved. First, it is currently elusive how different environmental stimuli induce appropriate electrical signals with unique phase signatures. Functional and mechanical studies on ion channels and pumps that shape dynamics of SWPs would be required. Second, given that Glu-mediated activation of GLR ion channels plays a central role in the generation and systemic propagation of SWPs after wounding, it should be addressed whether subcellular localization of GLRs is related to the systemic signal propagation. Several GLR proteins are predicted to be targeted to the endoplasmic reticulum and play a potential function in secretory pathway (Davenport, 2002; Bassham et al., 2008; Nguyen et al., 2018), as shown by the rice GLR3.1 protein (Li et al., 2006). Localization-dependent GLR functions should be investigated to understand a possible linkage of intracellular and intercellular communications. Third, although wounding increase apoplastic Glu in local injured tissue, it should be answered how it is conveyed to the distal parts of the plant (Qiu et al., 2019). Additionally, a range of cellular Glu concentrations that trigger systemic calcium and electrical waves *via* GLR proteins should also be elucidated.

The SWP contains information about the severity of wounding and the distance from injured sites. Based on the information, plants induce appropriate wound responses by regulating intracellular Ca^2+^ concentration and endogenous JA levels in local and systemic tissues. Since current studies on wound-responsive electrical signals focus mainly on systemic aspects of defense responses and there is limited understanding on potential impact of SWPs in tissue healing and regeneration and pathogen resistance in local tissues (Kral et al., 2016; Hernandez-Coronado et al., 2022), it would be necessary to unravel the relevance of electrical signals in wound responses in local tissues in the near future.

The electrical signal intricately interacts with other systemic signals. Hydraulic signals contribute to propagation of SWP (Stahlberg and Cosgrove, 1996; Farmer et al., 2020). Altered hydrostatic pressure by wounding activates mechanosensitive elements that in turn affect membrane depolarization in systemic tissues (Moe-Lange et al., 2021). Similarly, ROS-dependent long-distance signaling also participates in SWP propagation (Farmer et al., 2020). Treatment with the NAD(P)H oxidase inhibitor, diphenyleneiodonium, reduces SWP duration in systemic tissues (Mousavi et al., 2013), indicating that ROS signaling potentially regulates SWP. Moreover, Ca^2+^ and SWP waves are also interdependent on each other. Despite the extensive connections among systemic signals, the molecular mechanisms underlying these interactions have been limitedly unveiled. It will be needed to identify molecular factors that are responsible for the crosstalk of systemic signals and ultimately comprehensive mechanisms that coordinate systemic signals to further understand efficiency and robustness of long-distance wound responses.

## Author Contributions

All authors listed have made a substantial, direct, and intellectual contribution to the work, and approved it for publication.

## Funding

This work was supported by the Basic Science Research (NRF-2021R1I1A1A01054378 to KL; NRF-2022R1A2B5B02001266 to PJS) and Basic Research Laboratory (NRF-2020R1A4A2002901) programs funded by the National Research Foundation of Korea and by the Creative-Pioneering Researchers Program through Seoul National University (0409–20210288).

## Conflict of Interest

The authors declare that the research was conducted in the absence of any commercial or financial relationships that could be construed as a potential conflict of interest.

## Publisher’s Note

All claims expressed in this article are solely those of the authors and do not necessarily represent those of their affiliated organizations, or those of the publisher, the editors and the reviewers. Any product that may be evaluated in this article, or claim that may be made by its manufacturer, is not guaranteed or endorsed by the publisher.
